# Exosomes: compositions, biogenesis, and mechanisms in diabetic wound healing

**DOI:** 10.1186/s12951-024-02684-1

**Published:** 2024-07-05

**Authors:** Yichuan Li, Zhanyong Zhu, Sicheng Li, Xiaohang Xie, Lei Qin, Qi Zhang, Yan Yang, Ting Wang, Yong Zhang

**Affiliations:** 1 Department of Dermatology, Tongji Hospital, Tongji Medical College, Huazhong University of Science and Technology, Wuhan, 430030 China; 2 Department of Plastic Surgery, Renmin Hospital of Wuhan University, Wuhan, Hubei Province 430060 China; 3 Department of Plastic and Cosmetic Surgery, Tongji Hospital, Tongji Medical College, Huazhong University of Science and Technology, Wuhan, Hubei 430030 China; 4 Xianning Medical College, Hubei University of Science & Technology, Xianning, Hubei 437000 China; 5 Health Management Center, Tongji Hospital, Tongji Medical College, Huazhong University of Science and Technology, Wuhan, 430030 China; 6 Department of Medical Ultrasound, Tongji Hospital of Tongji Medical College of Huazhong, University of Science and Technology, Wuhan, 430030 China

**Keywords:** Diabetes, Exosomes, MSCs, ncRNAs, Regeneration, Immune regulation

## Abstract

Diabetic wounds are characterized by incomplete healing and delayed healing, resulting in a considerable global health care burden. Exosomes are lipid bilayer structures secreted by nearly all cells and express characteristic conserved proteins and parent cell-associated proteins. Exosomes harbor a diverse range of biologically active macromolecules and small molecules that can act as messengers between different cells, triggering functional changes in recipient cells and thus endowing the ability to cure various diseases, including diabetic wounds. Exosomes accelerate diabetic wound healing by regulating cellular function, inhibiting oxidative stress damage, suppressing the inflammatory response, promoting vascular regeneration, accelerating epithelial regeneration, facilitating collagen remodeling, and reducing scarring. Exosomes from different tissues or cells potentially possess functions of varying levels and can promote wound healing. For example, mesenchymal stem cell-derived exosomes (MSC-exos) have favorable potential in the field of healing due to their superior stability, permeability, biocompatibility, and immunomodulatory properties. Exosomes, which are derived from skin cellular components, can modulate inflammation and promote the regeneration of key skin cells, which in turn promotes skin healing. Therefore, this review mainly emphasizes the roles and mechanisms of exosomes from different sources, represented by MSCs and skin sources, in improving diabetic wound healing. A deeper understanding of therapeutic exosomes will yield promising candidates and perspectives for diabetic wound healing management.

## Introduction

**Fig. 1:**
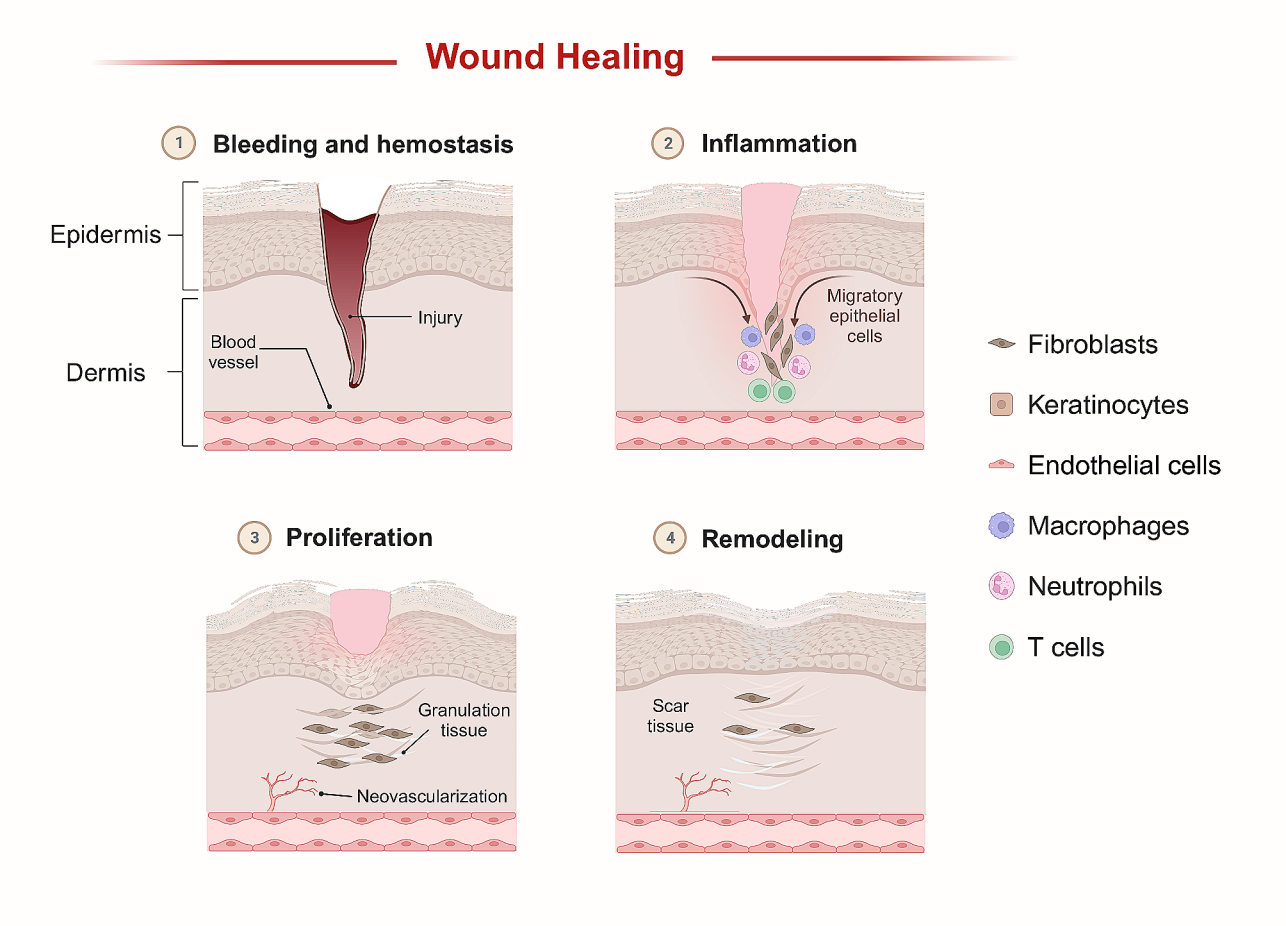
Wound healing phases. Wound healing is a remarkably sophisticated biological process that can be categorized into four chronologically sequential and overlapping phases including hemostasis, inflammation, proliferation, and remodeling. Multiple cell-secreted factors and cells within the wound lesion, mainly including fibroblasts, keratinocytes, endothelial cells, and immune cells, are critical for remodeling wound healing. Platelets are pivotal in maintaining the hemostatic phase. Immune cells play an anti-infective role mainly during the inflammatory phase. Cell proliferation, migration, re-epithelialization, neovascularization, and extracellular matrix production are key biological events during these phases. During the remodeling phase, collagen I gradually replace collagen III in the wound site. It was created by taking the template on BioRender.com as a reference, with permission

Wound healing is a highly complex biological process involving the collaboration of multiple tissues and cells to repair and rebuild injured tissue structures and cells [1]. This process can be divided into four chronologically sequential and overlapping phases: hemostasis, inflammation, proliferation, and remodeling [2] **(**Fig. 1**)**. Due to various pathophysiologic symptoms, such as diabetic peripheral neuropathy, peripheral vascular disease, atherosclerosis, immunopathy, and neuroarthropathy, diabetes mellitus (DM) can cause incomplete or delayed wound healing [3, 4]. Diabetic wounds represent a significant therapeutic challenge due to their complicated features, including hyperglycemia, infection, chronic inflammation, impaired microcirculation, hypoxia, and impaired neuropeptide signaling. Current conventional clinical therapies, including glycemic control, surgical debridement, wound dressing, wound off-loading, vascular assessment, and infection control, have merits and limitations. Recently, exosomes, as mediators of cellular interactions and carriers of cellular signals, have emerged as noncellular therapeutic candidates due to their unique biological properties and therapeutic potential.

Exosomes are extracellular vesicles (EVs) with a bilayer lipid membrane structure, ranging in size from 30 to 150 nm [5]. Exosome formation can be divided into four distinct phases starting with deformation and invagination of the plasma membrane to form early endosomes. Early endosomes mature and develop into multivesicular bodies (MVBs). Subsequently, the MVBs generate intraluminal vesicles (ILVs) by inward sprouting, a process involving multiple mechanisms. The pathways of ILVs are primarily categorized into endosomal sorting complex required for transport (ESCRT)-dependent and ESCRT-independent mechanisms, with the ESCRT-dependent pathway containing ubiquitin-dependent and ubiquitin-independent subpathways. The ESCRT complex consists of ESCRT-0, ESCRT-I, ESCRT-II, and ESCRT-III, each of which has distinct biological functions [6]. Specifically, ESCRT-0 selectively binds ubiquitinated substrates through its ubiquitin-binding domain (UBD), the ESCRT-I and ESCRT-II complexes are responsible for driving the inward budding of the endosomal membrane, and the ESCRT-III complex mediates cargo segregation during ILV formation [7]. In addition, certain nonubiquitinated substrates can be sorted into ILVs via interactions with proteins such as Alix, Hrs, and syntenin-1, demonstrating the versatility of the ESCRT pathway in managing diverse intracellular cargo [8]. Studies have shown that ILVs can still be generated even when all key ESCRT-associated subunits are removed, suggesting the existence of ESCRT-independent mechanisms. For example, the lipid-modifying enzyme nSMase2 converts sphingomyelin into ceramides, which not only promotes the formation of lipid rafts but also triggers membrane invagination and outgrowth, thereby participating in ILV formation [9]. Moreover, tetraspanins, such as CD9, CD63, CD81, and CD82, could form tetraspanin-enriched microdomains (TEMs), and facilitate intracellular signaling, providing essential platforms for the sorting and clustering of proteins within exosomes [10]. Finally, a portion of MVBs fuse with lysosomes for degradation. Under the regulation of Rab proteins and SNARE complexes, another portion of MVBs fuse with the plasma membrane and release exosomes in the form of vesicles. These mechanisms collaborate to ensure the formation and secretion of exosomes.

**Fig. 2:**
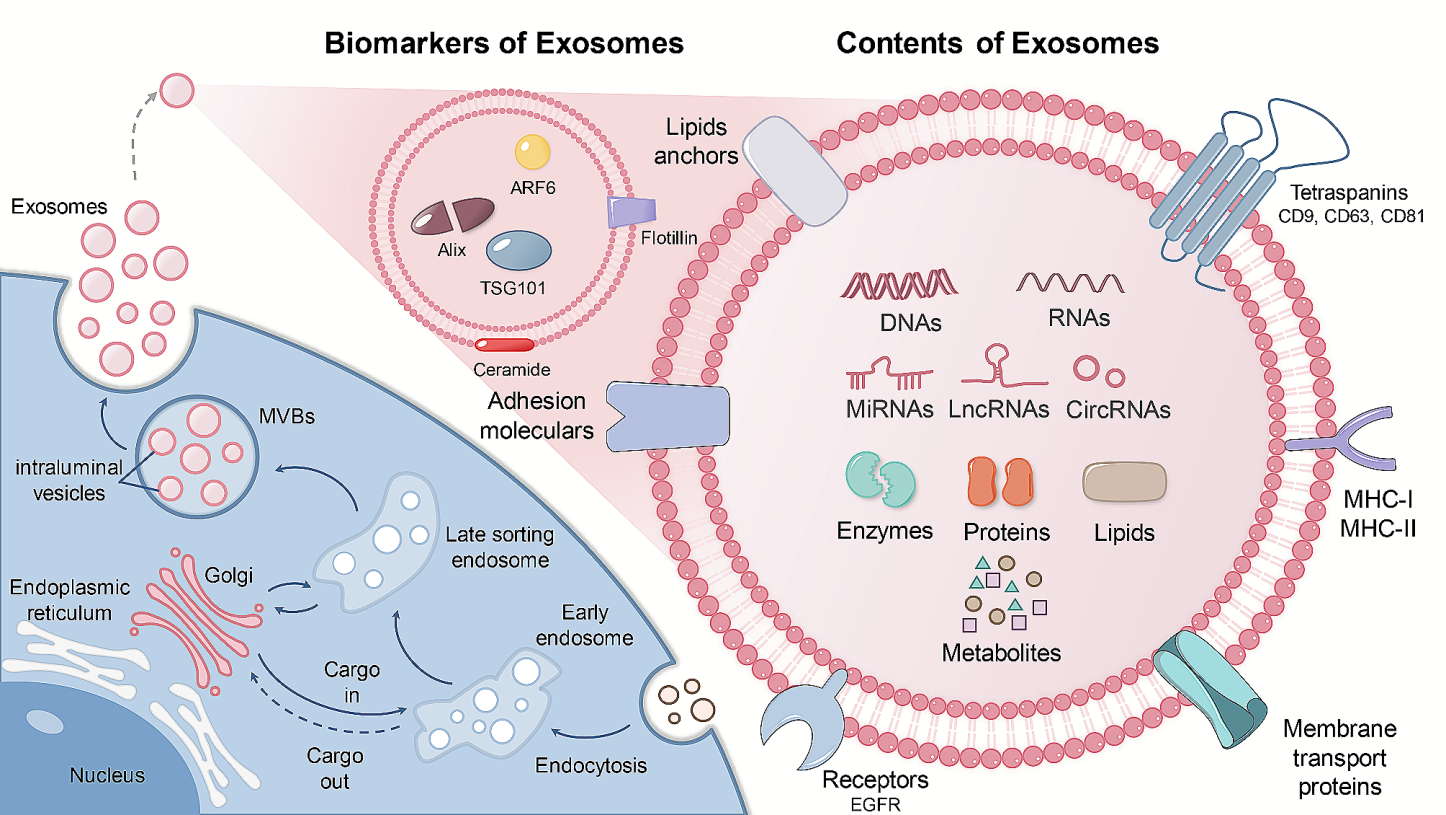
The biogenesis, identification, and components of exosomes. Various components of exosomes, including proteins, lipids, and molecule metabolites, are endocytosed to form early endosomes. Afterward, early endosomes are transformed into late endosomes, which subsequently form multivesicular bodies (MVBs). MVBs can fuse with the plasma membrane through a network of microtubules and cytoskeleton and release exosomes. Exosomes contain a variety of cellular components with a lipid bilayer structure and express a series of characteristic, conserved, key markers associated with exosome biosynthesis, including nucleic acids (DNAs, mRNAs, miRNAs, circRNAs, lncRNAs), biogenesis-related proteins (Alix, TSG101), membrane transport and fusion proteins (Annexin, Rab5), transmembrane proteins (CD9, CD63, CD81), antigen presentation (MHC-I and MHC-II), adhesion molecules, receptors (EGFR), enzymes (GAPDH, ATPase), lipids, metabolites. MVBs, multivesicular bodies; MHC, major tissue compatibility complexes

Once released into the extracellular space, exosomes can enter the target cell membrane through membrane fusion, endocytosis, or ligand-receptor interactions [11]. Membrane fusion allows exosomes to directly release their contents into the cytoplasm of target cells. Endocytosis is the primary mode of exosomal cellular entry and operates through various processes, including clathrin-mediated endocytosis, caveolin-mediated endocytosis, macropinocytosis, and phagocytosis [12]. After endocytosis, exosomes fuse with endosomal membranes and then release their cargo into the cytoplasm to exert their effects through a process known as endosomal escape. If exosomes fail to escape from endosomes, their cargo may be degraded by lysosomes or resecreted outside the cell, failing to impact the target cell as intended. Through the ligand-receptor model, surface molecules of exosomes, including fibronectins, tetraspanins, proteoglycans, lectin receptors, and immunoglobulins, bind to receptors on target cells, triggering activation of downstream signaling pathways and regulating gene expression. The process of cellular uptake and utilization of exosomes is influenced by various factors; notably, the local acidic microenvironment significantly enhances the release of exosomes and their binding efficiency to target cells [13]. The low pH of endosomes enhances the efficiency of exosome release into the cytoplasm, while cholesterol accumulation in endosomes inhibits this process [14]. Exosomes are widely distributed in different body fluids, such as blood, urine, milk, amniotic fluid, saliva, semen, and cerebrospinal fluid. Therefore, exosomes are recognized as valuable messengers for liquid biopsies of various diseases. By transferring biologically active components, such as nucleic acids, proteins, lipids, and multimolecular complexes, exosomes can mediate intercellular communication and intercellular exchange of substances to regulate metabolism and improve wound healing **(**Fig. 2**)**. Currently, exosomes have been exploited as drug delivery vehicles for treating various diseases, with the merit of low immunogenicity, low cytotoxicity, low tumorigenicity, low degradation, high stability, ease of preservation, and ability to penetrate the blood-brain barrier [15].

The specific functions of exosomes depend on their origins, as exosomes are products and reflections of their parent cells. For instance, in the context of diabetic wound healing, both adipose-derived stem cell-derived exosomes (ADSC-exos) and bone marrow mesenchymal stem cell-derived exosomes (BMSC-exos) exhibit overlapping functions, exerting positive effects on fibroblasts (FBs), keratinocytes (KCs), and endothelial cells (ECs). Nevertheless, ADSC-exos showed superior efficacy and rapidity in promoting diabetic wound healing compared to BMSC-exos. The cargo carried by ADSC-exos is primarily responsible for angiogenic functions, while the cargo carried by BMSC-exos is mainly associated with cell proliferation and viability [16]. The placenta serves as a reservoir of stem cells, providing various types of placental mesenchymal stem cells (PMSCs) that provoke less ethical controversy, such as human umbilical cord mesenchymal stem cells (hucMSCs), human amniotic mesenchymal stem cells (hAMSCs), and human amniotic epithelial cells (hAECs). Compared to BMSCs, PMSCs have a greater proliferative capacity and secrete more types of cytokines and growth factors to preserve cellular mitochondrial function [17–19]. Exosomes derived from PMSCs show distinct therapeutic potential in regulating different aspects of diabetic wound healing. Notably, hucMSC-exos modulate macrophage polarization, attenuate oxidative stress, and inflammation, and accelerate diabetic wound healing. hAMSC-exos improve EC function and promote angiogenesis in HG environments through enriched lncRNAs. hAEC-exos markedly promoted the activities of human umbilical vein endothelial cells (HUVECs) and human fibroblasts (HFBs), contributing significantly to the acceleration of diabetic wound healing. Exosomes are derived from skin cellular components and can modulate inflammation and promote the repair and regeneration of key skin cells, which in turn promotes skin healing. For instance, as a major component of the skin epidermis, KCs serve as the primary line of defense against skin injury and are involved in the re-epithelialization process [20]. Skin injury significantly induces KCs to release exosomes. KC-derived exosomes (KC-exos) mediate communication between KCs and macrophages, modulating inflammation and expediting wound closure [21]. Dermal fibroblasts (DFs) can secrete significant quantities of collagen, contributing to the production of the extracellular matrix (ECM), which plays a crucial role in skin remodeling. DF-derived exosomes (DF-exos) promote wound collagen deposition, re-epithelialization, and angiogenesis, thereby facilitating diabetic wound healing.

**Fig. 3:**
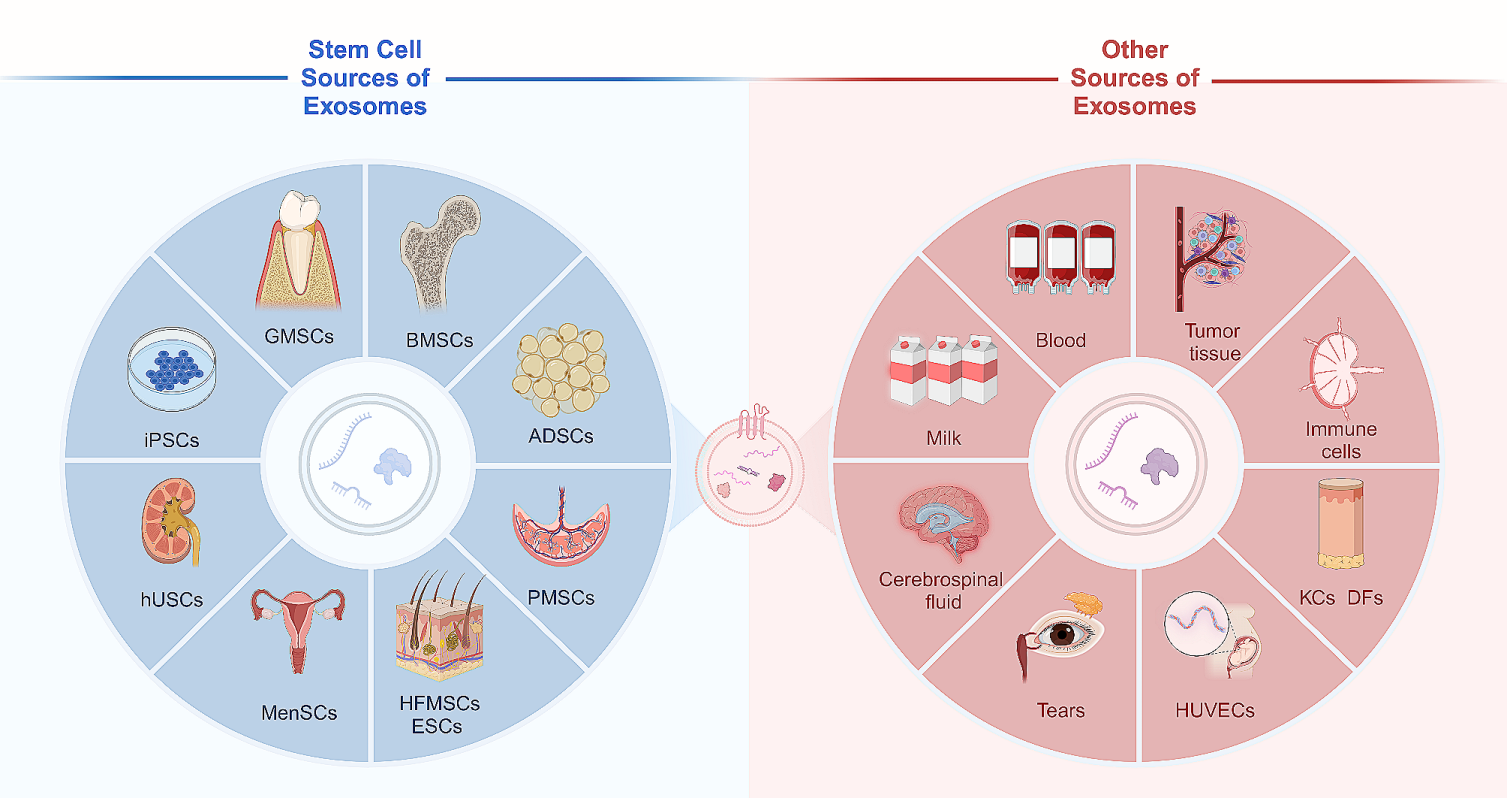
Stem cell sources and other sources of exosomes for diabetic wound healing. Exosomes derived from MSCs have superior potential in the field of diabetic wound healing, including BMSCs ADSCs, PMSCs, HFMSCs, ESCs, MenSCs, hUSCs, iPSCs, and GMSCs. Besides, Other sources of exosomes are also potential candidates for wound healing, including blood, milk, cerebrospinal fluid, tears, tumor tissue, immune cells, keratinocytes, dermal fibroblasts, and HUVECs. It was created with BioRender.com

Therefore, this review mainly describes the roles and mechanisms of exosomes from different sources, namely, MSCs and skin sources, in improving diabetic wound healing **(**Fig. 3**)**. A deeper understanding of therapeutic exosomes will yield promising candidates and perspectives for diabetic wound healing management.

## ADSC-exos

ADSCs, which are abundant in adipose tissue, are characterized by easy accessibility, high proliferative potential, and strong self-renewal ability [22]. Moreover, ADSCs exhibit multilineage differentiation potential, with the ability to differentiate into adipocytes, chondrocytes, osteocytes, dermal tissues, and neural tissues, reflecting their possible value in various tissue repair processes [23]. ADSCs can secrete cytokines, growth factors, and exosomes through paracrine secretion to promote wound healing and tissue regeneration. Exosomes constitute one of the primary components of paracrine secretion and a major contributor to the actions of ADSCs. ADSC-exos are rich in microRNAs (miRNAs), long-stranded noncoding RNAs (lncRNAs), circular RNAs (circRNAs), and functional proteins. In addition, ADSC-exos can alleviate the functional inhibition of KCs, ECs, human dermal fibroblasts (HDFs), endothelial progenitor cells (EPCs), and HUVECs in a high-glucose (HG) environment. ADSC-exos also modulate the immune response, promote vasculogenesis, accelerate skin cell proliferation and re-epithelialization, and regulate collagen remodeling to promote diabetic wound healing [24].

### Pretreatment with ADSC-exos

Oxidative stress is tightly associated with the initiation and development of diabetic wound healing. Under diabetic conditions, localized hypoxia and HG lead to high ROS production by mitochondria, contributing to vascular cell damage and inflammation [25]. SIRT3 is one of the most essential deacetylases and modulates the level of acetylation in mitochondria and decreases the accumulation of intracellular ROS [26]. ADSC-exos increased the activity of SOD2 by upregulating SIRT3, thereby decreasing the levels of inflammatory factors and mitochondrial oxidative stress in HUVECs [27]. Furthermore, ADSC-exos decreased ROS production in KCs, ECs, and FBs, which was mediated by activation of the eHSP90/LRP1/Akt signaling pathway [28]. Taken together, these findings indicate that ADSC-exos have a strong potential for oxidative stress regulation.

Studies have shown that pretreated ADSCs display enhanced paracrine effects. In hypoxic environments, ADSCs secrete more exosomes, which improves blood perfusion and graft tissue survival and reduces inflammation [29]. Wang et al. reported that hypoxic adipose stem cell-derived exosomes (HypADSC-exos) could facilitate proliferation, migration, vascular growth factor secretion and ECM remodeling via activation of the PI3K/Akt pathway in HFBs [30]. Accordingly, HypADSC-exos accelerated re-epithelialization and wound closure in diabetic nude mice.

### NcRNAs from ADSC-exos

Noncoding RNAs (ncRNAs) mainly consist of miRNAs, lncRNAs, and circRNAs. NcRNAs function as modulators capable of regulating transcription and translation of the genome at the RNA level [31]. Exosomally delivered ncRNAs play a vital role in diabetic wound healing by regulating immune responses and cellular functions, thus serving as markers for the clinical diagnosis of DM.

#### MiRNAs from ADSC-exos

MiRNAs are a group of highly conserved small ncRNAs with a molecular length of 18–25 nucleotides that regulate cellular biological behaviors, such as angiogenesis, and immune responses through targeted binding to downstream miRNA untranslated regions (UTRs) [32]. MiRNAs are key messengers for intercellular communication. After miRNA-loaded ADSC-exos are absorbed by target cells, ADSC-exos can regulate the expression of corresponding genes and proteins in cells through the specific regulation of miRNAs, thus improving the healing of diabetic wounds.

Engineered miR-21-5p-abundant hADSC-exos (miR-21-5p-exos) were obtained by Lv et al. by loading miR-21-5p mimics into hADSC-exos via electroporation [33]. MiR-21-5p-exos promoted the proliferation and migration of KCs in vitro by stimulating the Wnt/β-catenin signaling pathway and promoted diabetic wound healing in vivo by accelerating re-epithelialization, collagen remodeling, angiogenesis, and vessel maturation.

Survival of randomized flap grafts is substantially reduced due to the severe inflammatory response and limited cell proliferation around diabetic wounds. Ge et al. generated engineered miR-132-abundant ADSC-exos (miR-132-exos) by lentiviral transduction of ADSCs [34]. In vivo, miR-132-exos markedly improved flap survival and promoted diabetic wound healing by attenuating local inflammation and stimulating NF-κB-mediated M2 macrophage polarization. The intrinsic therapeutic potential of ADSC-exos was enhanced by altering miRNA expression in exosomes through electroporation and lentiviral transduction techniques. In addition, compared to stem cell-derived exosomes, these engineered exosomes are characterized by high yield, high purity, and high targeting capabilities, which makes them promising materials for clinical application [35].

#### LncRNAs from ADSC-exos

LncRNAs are poorly conserved ncRNAs measuring more than 200 nucleotides in length and can regulate epigenetic modifications and transcriptional and posttranscriptional processes [36]. As competitive endogenous RNAs, lncRNAs bind to the complementary binding site of miRNAs and then control various signaling pathways by sponging the 3’-UTRs of downstream target genes [37].

Linc00511 modulates the growth and migration of cancer cells and the expression of vascular endothelial growth factor (VEGF) and angiogenesis [38]. Qiu et al. found that exosomes from linc00511-overexpressing ADSC-exos facilitated wound healing in diabetic rats and proliferation, migration, and angiogenesis in EPCs by suppressing PAQR3-induced ubiquitin-mediated degradation of Twist1 [39]. These findings provide the basis for the future clinical use of ADSC-exos combined with artificially modified lncRNAs to accelerate wound healing in diabetic patients.

#### CircRNAs from ADSC-exos

CircRNAs, a class of noncoding RNAs with covalent closed-loop structures, can regulate gene expression by inhibiting miRNA activity, thus playing roles in physiological processes such as the cell cycle and aging [40]. CircRNAs are important diagnostic biomarkers of diabetic wound healing [41–43].

As a pivotal nicotinamide adenosine dinucleotide (NAD)-dependent deacetylase, SIRT1 functions as a crucial modulator of metabolism and aging by inhibiting the acetylation of FOXO1, a crucial negative regulator of angiogenic activity [44]. Wang et al. found the upregulation of circ-Astn1 expression in ADSC-exos [45]. Under HG conditions, circ-Astn1 overexpression in ADSC-exos restored angiogenesis and inhibited the apoptosis of EPCs by inhibiting the miR-138-5p/SIRT1/FOXO1 axis. Shi et al. demonstrated that overexpression of mmu_circ_0000250 in ADSC-derived exosomes activated autophagy and thus increased angiogenesis in EPCs under HG conditions to accelerate diabetic wound healing by upregulating SIRT1 through the absorption of miR-128-3p [46]. In addition, they also found that circ-Snhg11 in ADSC-exos suppressed HG-induced EC injury and induced M2-like macrophage polarization through the miR-144-3p/HIF-1α axis [47].

FGFs primarily function in mitosis, development, transformation, angiogenesis, and survival [48]. FGF4 promotes p38 MAPK/GSK3β-mediated Slug stabilization, which triggers epithelial-to-mesenchymal transition (EMT) and promotes KC migration and proliferation, thereby facilitating wound healing [49]. Liang et al. revealed that the ADSC-derived exosomal mmu_circ_0001052 accelerated HUVEC angiogenesis by binding to miR-106a-5p, which was followed by activation of the FGF4/p38 MAPK pathway, to ameliorate the effects of the HG environment [50]. This effect drove the vascularization of HUVECs and accelerated the closure of diabetic wounds.

### Proteins from ADSC-exos

Modification of exosomes using bioengineering techniques allows the isolation of higher concentrations of exosomes and the construction of more stable particles with specific therapeutic functions, thereby improving the targeting ability of natural exosomes and prolonging their onset of action [51]. Transcription factor gene modification is a strategy for exosome engineering that regulates gene expression and thus influences cellular behavior and physiological responses. Moreover, combining transcription factors with miRNAs enhances wound healing by enabling precise control over cellular communication, significantly boosting the functional efficacy of exosomes.

NFIC is a nuclear factor I family member that affects cell growth and differentiation by binding to the conserved N-terminal DNA-binding domain and exerting transcriptional control on target genes [52]. NFIC has been implicated in the regulation of diabetes-related diseases such as diabetic nephropathy. Huang et al. found that NFIC overexpression of hADSC-exos upregulated the level of miR-204-3p and thus inhibited the level of HIPK2, thereby alleviating the inhibition of HG-induced proliferation, migration, and angiogenesis in HUVECs [53].

Furthermore, the nuclear factor red factor 2-related factor (Nrf2) is a transcription factor that functions as a major regulator of redox homeostasis in cells [54–56]. Li et al. demonstrated that the Nrf2-overexpression of ADSC-exos alleviated stress-induced premature senescence of EPCs in an HG environment by facilitating the expression of SMP30, VEGF, and VEGFR2 phosphorylation, with the suppression of ROS and inflammatory cytokines [57]. In a diabetic rat model, Nrf2-overexpression of ADSC-exos increased granulation tissue formation and angiogenesis and decreased inflammation and oxidative stress-related proteins to accelerate diabetic wound healing.

Similarly, IRF1 is a nuclear transcription factor that mediates interferon-associated antiviral and antitumor responses [58]. IRF1 reportedly accelerates diabetic wound healing by directly binding to the iNOS promoter to induce angiogenesis [59]. Wu et al. found that IRF1 overexpression-modified exosomes from ADSCs accelerated FB proliferation and migration and promoted rapid healing of foot skin wounds in diabetic rats, effects that were accompanied by increased vascularization of ECs [60]. Specifically, IRF1 bound to miR-16-5p and inhibited the expression of its target gene SP5.

In summary, ADSC-exos accelerate diabetic wound healing by activating multiple signaling pathways, such as the SIRT3/SOD2, eHSP90/LRP1/Akt, PI3K/Akt, Wnt/β-catenin, NF-κB, FGF4/p38MAPK, and miR-138-5p/SIRT1/FOXO1 pathways. These comprehensive effects highlight the multifunctionality and effectiveness of ADSC-exos in regulating diabetic wound healing, especially by reducing oxidative stress and inflammation and promoting angiogenesis.

## BMSC-exos

BMSCs are self-renewing and pluripotent stem cells that can differentiate into osteoblasts, chondrocytes, adipocytes, and even myoblasts, making them an ideal source of cells for treating multiple diseases [61]. BMSCs can directly participate in tissue repair by differentiating into various cells. However, transplantation therapy with BMSCs involves challenges in terms of homing, survival, and immune rejection [62]. In addition to differentiating into various cellular lineages, paracrine signaling by BMSCs is also critical for inflammatory responses, fibrosis, apoptosis, promotion of angiogenic responses, and immune regulation, thereby improving the microenvironment to accelerate the recovery of damaged tissues. Compared to BMSCs, BMSC-exos exhibit superior stability, permeability, biocompatibility, and immunomodulatory capabilities [63]. For instance, BMSC-exos enhanced EPC tube formation and inhibited inflammation in diabetic wounds by upregulating Nrf2 and subsequently downregulating TNF-α and IL-1β [64]. In addition, the combination of BMSC-exos and Nrf2 activators showed a stronger effect on re-epithelialization, vascularization, and collagen deposition in diabetic wounds. Furthermore, BMSC-exos activate the Akt, Erk1/2, and STAT3 signaling pathways, thereby inducing the expression of IL-6 and insulin-like growth factor-1 (IGF1) to accelerate vascularization and epithelialization [65].

### Pretreatment with BMSC-exos

Previous studies have shown that pretreatment of MSCs with physical, chemical, and biological factors is an effective method for enhancing the bioactivity of MSCs and improving their repair efficacy in tissue engineering and regenerative medicine [66]. PTEN is a dual phosphatase that antagonizes PI3K activity and negatively regulates the PI3K/Akt/eNOS signaling pathway [67, 68]. The activation of the PI3K/Akt/eNOS pathway upregulates VEGF and accelerates diabetic wound healing by promoting cell proliferation, migration, maintenance of vascular homeostasis, and collagen synthesis [69].

Atorvastatin (ATV) accelerated tissue repair in acute injuries and the expression of proteins and cytokines associated with cell growth in rats, which was independent of its lipid-lowering effects. Yu et al. identified that ATV-preconditioned BMSC-exos (ATV-exos) enhanced the activities and significantly upregulated VEGF, PDGF, EGF, bFGF, and ANG1 in HUVECs by upregulating miR-221-3p, inhibiting PTEN, and subsequently activating the Akt/eNOS pathway, thereby accelerating blood vessel formation to facilitate wound healing [70].

Pioglitazone (PGZ), a peroxisome proliferator-activated receptor activator, is a commonly used drug for the treatment of DM because of its anti-inflammatory and antioxidant effects. Hu et al. elucidated that PGZ-pretreated BMSC-exos (PGZ-exos) facilitate the angiogenic function of HUVECs and potentiate vascularization and ECM remodeling, thus accelerating diabetic wound healing in rats via inhibition of the PTEN/PI3K/Akt/eNOS pathway [71].

Previous studies have found that by transporting adipose-derived exosomes to macrophages, melatonin (MT) can increase the proportion of M2 macrophages and alleviate adipose inflammation [72]. Liu et al. explored that MT-pretreated BMSC-exos (MT-exos) increased M2 polarization and suppressed inflammatory responses by activating the PTEN/Akt signaling pathway [73]. Furthermore, MT-exos markedly increased the levels of α-smooth muscle actin (α-SMA), collagen I, and collagen III in vivo, which accelerated angiogenesis and collagen synthesis to facilitate diabetic wound healing.

### NcRNAs from BMSC-exos

#### MiRNAs from BMSC-exos

MiR-155 impairs diabetic wound re-epithelialization by targeting FGF7, which has antimicrobial properties and is essential for perfusion and wound closure [74]. Gondaliya et al. clarified that the BMSC-exos loaded with miR-155 inhibitor promoted the expression of FGF-7 and VEGFA and downregulated the expression of MMP-2, MMP-9, and inflammatory factors in HaCaTs, which accelerated angiogenesis and re-epithelialization and ultimately improved wound healing [75]. This study revealed that BMSC-exos loaded with miR-155 inhibitors could be a promising clinical option for accelerating wound healing in patients with diabetes.

Lu et al. identified that miR-126-3p enriched in IFN-γ-pretreated BMSC-exos (I-exos) significantly promoted angiogenesis in vivo and ex vivo, subsequently improving diabetic wound healing by binding to SPRED1 and activating the Ras/Erk signaling pathway [76]. SPRED1 is a therapeutic target for vascular endothelial cell repair in diabetes.

#### LncRNAs from BMSC-exos

Han et al. identified that BMSC-derived exosomal lncRNA KLF3-AS1 facilitated the activity of HUVECs under HG conditions by downregulating miR-383 and upregulating its target VEGFA [77]. These effects were favorable for stimulating angiogenesis and accelerating diabetic wound healing in vivo.

Li et al. demonstrated that lncRNA H19 from BMSC-exos enhanced FB proliferation and migration and inhibited apoptosis and inflammation by binding to miR-152-3p, subsequently activating the PTEN/PI3K/Akt axis [78]. In addition, overexpression of lncRNA H19 in BMSC-exos significantly increased the expression of VEGF, TGF-β1, α-SMA, and collagen I, which improved revascularization and accelerated diabetic wound closure.

#### CircRNAs from BMSC-exos

Ferroptosis is a type of regulated programmed cell death caused by iron overload and ROS-induced accumulation of lipid peroxides [79]. Ferroptosis plays a key role in the pathogenesis of diabetes and a variety of diabetes-related complications, such as diabetic nephropathy and diabetic cardiomyopathy [80]. Chen et al. illustrated that circ-ITCH-overexpressing BMSC-exos restored viability and angiogenic capacity and inhibited ferroptosis in HUVECs by recruiting the TAF15 protein and activating the Nrf2 signaling pathway, thus accelerating diabetic wound healing [81]. Circ-ITCH shows extraordinary potential in treating diabetic wounds.

The above studies underscore multiple strategies to enhance the function of BMSC-exos via pretreatment or engineering. We specifically focus on the PTEN/PI3K/Akt signaling pathway, a key mechanism that triggers diverse physiological responses in different cellular environments, such as HUVECs, M2 macrophages, and FBs, highlighting the importance of precise regulatory strategies.

## PMSC-exos

The placenta, a highly vascularized organ of maternal origin, is usually discarded after delivery. Therefore PMSCs can be obtained in a non-invasive manner and are free from ethical and legal constraints [82]. These PMSCs possess robust self-renewal capabilities, pluripotency, and immunomodulatory functions and are associated with a low risk of viral contamination. Compared with BMSCs, ucMSCs, and ADSCs, PMSCs exhibit significant immunomodulatory advantages, especially after IFN-γ stimulation, and the MHC class II expression of PMSCs is minimally upregulated [83]. Furthermore, PMSCs can protect ECs under conditions of oxidative stress and inflammation, highlighting their therapeutic potential in diabetic wounds [84]. In addition, PMSCs can be assumed to have greater angiogenic potential as they reside predominantly in the vascular wall niches of the placenta [85]. PMSCs include multiple MSCs derived from different parts of the placenta, such as hucMSCs, hAMSCs, and hAECs.

### hucMSC-exos

hucMSCs are typical adult stem cells with distinct advantages in terms of sufficient supply, painless collection, and rapid self-renewal [86]. hucMSCs can synthesize and secrete different trophic factors and cytokines to enhance the expansion and function of hematopoietic stem cells, embryonic stem cells, and natural killer cells. hucMSC-derived exosomes (hucMSC-exos) can ameliorate oxidative stress injury and the inflammatory response induced by HG environment in HUVECs [87]. In addition, hucMSC-exos accelerated angiogenesis and granulation tissue production to boost diabetic wound healing.

hucMSC-exos can induce macrophage M2 polarization, which downregulates TNF-α to inhibit inflammatory infiltration, accelerates neoangiogenesis by upregulating CD31 and VEGF, and promotes collagen synthesis in vivo [88]. Furthermore, hucMSC-exos promoted the proliferation of HUVECs and NIH-3T3 cells in vitro and therefore promoted diabetic wound healing.

#### Pretreatment with hucMSC-exos

Nocardia rubra cell wall skeleton (Nr-CWS) has been found to activate macrophages, increase TGF-β1 expression, and promote angiogenesis as a biological response modifier, resulting in accelerated skin wound healing. Li et al. demonstrated that Nr-CWS-pretreated hucMSC-exos possessed a significant proangiogenic effect by upregulating VEGF, bFGF, and HGF in vitro and in vivo, thereby accelerating diabetic wound healing mediated by activating the circIARS1/miR-4782-5p/VEGFA axis [89].

Furthermore, LPS pretreatment has been reported to significantly enhance the paracrine function of ADSCs and to enhance the regenerative and reparative properties of tissues [90]. The let-7 miRNA family functions in the immune response and modulates inflammation [91]. Ti et al. showed that miRNA let-7b-enriched LPS-preconditioned hucMSC-exos (LPS-exos) promoted the transformation from THP-1 cells to M2 macrophages, thus upregulating the expression of anti-inflammatory cytokines, which facilitated wound healing [92]. In terms of mechanism, these effects were mediated by inhibition of the TLR4/NF-κB/STAT3/Akt signaling pathway. Pretreatment of hucMSC-exos with Nr-CWS and LPS could upregulate the expression of proangiogenic and anti-inflammatory mediators by controlling their respective specific miRNAs to further enhance their wound healing ability.

#### CircRNAs from hucMSC-exos

circHIPK3 has been proven to be an important factor in diabetes and diabetic complications, such as diabetic nephropathy, diabetic retinopathy, and diabetic cardiomyopathy. Liang et al. found that circHIPK3-overexpressing hucMSC-exos accelerated vascularization for diabetic wound healing in vivo by downregulating miR-20b-5p to upregulate Nrf2 and VEGFA [93].

#### Proteins from hucMSC-exos

NO in diabetic wound healing improves collagen remodeling and repairs wound mechanical strength. eNOS is one of the enzymes responsible for NO synthesis, and the concentration of eNOS at the wound site affects the rate of wound closure, the strength of the wound, and the growth of capillaries [94]. Zhao et al. illustrated that the hucMSC-exos loaded with large amounts of eNOS by genetic engineering and optogenetic techniques (hucMSC-exos/eNOS) could accelerate wound vascularization by activating the PI3K/Akt/mTOR or Fak/Erk1/2 signaling pathways [95]. Additionally, hucMSC-exos/eNOS promoted autophagy, M2 polarization, Treg cell aggregation, and TRM cell residency, thus modulating the immune microenvironment for diabetic wound healing.

In summary, hucMSC-exos can accelerate diabetic wound healing by activating signaling pathways, such as circIARS1/miR-4782-5p/VEGFA, PI3K/Akt/mTOR, and Fak/Erk1/2, or by inhibiting the TLR4/NF-κB/STAT3/Akt pathway.

### hAMSC-exos

hAMSCs are derived from the avascular amnion layer of the placenta, a transparent, smooth, single-layer membrane that covers the fetus and holds the amniotic fluid. hAMSCs can be induced to differentiate into osteoblasts, adipocytes, or endothelial cells and express a variety of stem cell markers, such as Oct-4 and Nanog, demonstrating their essential stem cell characteristics. Moreover, hAMSCs can inhibit activation of multiple immune cells, increasing the potential for treating immune-mediated diseases and attenuating transplant rejection [96]. In an in vivo study of hindlimb ischemia, implantation of hAMSCs increased blood perfusion and capillary density, suggesting that hAMSCs have potent angiogenic properties [97]. In addition, the conditioned medium of hAMSCs contains abundant anti-inflammatory and tissue-repairing factors that protect human KCs and DFs to facilitate re-epithelialization in wounds, suggesting a significant paracrine effect [98]. Fu et al. identified that hAMSC-derived exosomes (hAMSC-exos) alleviated the inhibition of proliferation, migration, and tube formation of HUVECs in HG environments [99]. In addition, hAMSC-exos were enriched with multiple angiogenesis-related lncRNAs, such as PANTR1 and H19, and thus accelerated angiogenesis and promoted diabetic wound healing.

### hAEC-exos

hAECs are derived from the human amniotic membrane and originate from the ectoderm during embryonic development. hAECs have low immunogenicity and anti-inflammatory properties and do not lead to teratomas after transplantation, which makes hAECs an ideal source for clinical cell transplantation and regenerative medicine [100]. hAEC-derived exosomes (hAEC-exos) can exert antifibrotic, immunomodulatory, and tissue regenerative effects, and also ameliorate wound healing and inhibit scar formation [101]. Wei et al. demonstrated that hAEC-exos facilitated angiogenic activity in HUVECs and increased the proliferative, migratory, and collagen deposition capacities of HFBs [102]. hAEC-exos also promoted diabetic wound healing by increasing the capillary density by activating the PI3K/Akt/mTOR pathway.

## Exosomes from skin tissues

### ESC-exos

Epidermal stem cells (ESCs) are a type of pluripotent cell attached to the basement membrane of the skin and are dedicated to the formation, differentiation, and regeneration of a functional epidermis [103]. Additionally, ESCs are highly malleable, readily available, and free of potential ethical issues. A clinical study demonstrated that epidermal basal cell suspensions containing ESCs improved wound healing in diabetic patients [104]. Compared to the features of ESCs, ESC-derived exosomes (ESC-exos) possess lower immunogenicity and are more stable under preservation and storage conditions.

ESC-exos contain significantly more miRNAs than FB-derived exosomes (FB-exos), as revealed by high-throughput sequencing [105]. Specifically, ESC-exos participate in homeostatic processes and cellular differentiation mediated by activating the TGFβ and PI3K/Akt signaling pathways. Furthermore, ESC-exos induced M2 macrophage polarization and accelerated FB and macrophage proliferation and migration, thus exerting anti-inflammatory and pro-vascularization effects on diabetic wound healing.

SOCS is a negative regulator of the JAK/STAT signaling axis. SOCS3 interferes with the proliferation and migration of KCs, thereby severely affecting the repair of the skin wound epithelium [106]. Yang et al. found that miR-203a-3p from ESC-exos promoted M2 macrophage polarization and the secretion of large amounts of VEGF, bFGF, and TGF-β by decreasing SOCS3 and activating the JAK2/STAT3 signaling pathway in macrophages in vitro, ultimately promoting diabetic wound healing [107]. In addition, they also illustrated that miR-200b-3p-rich ESC-exos could attenuate excessive autophagy-induced apoptosis of HUVECs in a HG environment by targeting the SYDE1/RAS/Erk pathway [108]. Furthermore, in vivo, miR200b-3p-rich ESC-exos accelerated diabetic wound healing by promoting vasculogenesis.

### HFMSC-exos

Hair follicles (HFs), which are important skin appendages, contain a variety of stem cells, such as melanocytes, epithelial stem cells, and MSCs [109]. HF-MSCs are mainly derived from dermal papillae-MSCs and dermal sheaths-MSCs, which possess multidimensional differentiation potential and the capacity to maintain skin homeostasis, and accelerate hair growth and regeneration [110]. In addition, HF-MSCs secrete paracrine factors to accelerate re-epithelialization and remodeling in diabetic wounds [111]. HF-MSC-derived exosomes (HF-MSC-exos) protect HDF viability, attenuate the impact of oxidative stress in hyperglycemia, and induce angiogenesis in HUVECs.

Pyroptosis is a novel type of programmed cell death characterized by cell swelling, pyroptosome formation, cell membrane rupture, and the release of large amounts of proinflammatory factors [112]. Activation of the NLRP3 inflammasome induces sustained inflammation, leading to cellular pyroptosis and subsequently impaired diabetic wound healing [113]. Yang et al. verified that exosomal lncRNA H19 from HFMSC-exos could promote proliferation and migration and inhibit the pyroptosis of HaCaTs by reversing the role of the NLRP3 inflammasome [114]. Through this effect, exosomal lncRNA H19 promoted diabetic skin wound healing.

### KC-exos

KCs, as the main component of the skin epidermis, are at the forefront of the innate immune response after skin injury. In addition, as a critical factor in epidermal reconstruction, KCs can repair the epidermal barrier through migration, proliferation, and differentiation for re-epithelialization [115]. Furthermore, KCs can recruit, activate, and regulate immune cells (ICs) by secreting cytokines, chemokines, and exosomes to reshape the immune state at wound sites.

#### Pretreatment with KC-exos

Aberrant interactions between skin cells contribute to the dysregulation of diabetic wounds. Fu et al. illustrated that LINC01435 from KC-exos pretreated with HG could upregulate HDAC8 by inducing the translocation of the YY1 into the nucleus, thereby suppressing tube formation and migratory capacity in HUVECs and ultimately impairing angiogenesis and diabetic wound healing [116]. Therefore, LINC01435 was expected to be a promising target for diabetic wound healing.

#### LncRNAs from KC-exos

TGFB1 and highly activated SMAD2/3 pathways benefit tissue fibrosis and tend to accelerate scar formation. Kuang et al. demonstrated that MALAT1-overexpressing KC-exos enhanced phagocytosis and M2 polarization and inhibited apoptosis in macrophages [117]. Specifically, KC-exos inhibited the ability of miR-1914-3p to activate MFGE8, thereby downregulating the TGFB1/SMAD3 signaling pathway. Ultimately, this effect facilitated diabetic wound healing and minimized scar formation.

### DF-exos

DFs located in the dermis of the skin are essential in skin remodeling and wound healing. DFs can synthesize and deposit ECM for skin-required elasticity and strength, thereby offering essential structural support for wound healing. During the proliferation phase of wound healing, DFs can transform into myofibroblasts with enhanced contractile capacity to facilitate wound contraction [118]. In addition, compared to other cell sources, DFs can be isolated from the skin by using less invasive procedures and are easier to culture. FB-exos can accelerate vascularization and collagen deposition, promoting the development of skin appendages and thereby accelerating wound healing [119]. Han et al. demonstrated that DF-exos preserved the features of HUVECs in HG environment in vitro, and promoted wound re-epithelialization, collagen deposition, and angiogenesis in vivo, thereby hastening diabetic wound healing [120]. These effects are mediated by DF-exos by activating the Akt/β-catenin pathway.

Skin tissues are primary responders to external irritations and infections, making their exosomes indispensable for the initial response to wounds. These exosomes not only participate directly in the natural repair processes of the skin, but are also closely associated with the structural reconstruction and functional recovery of the skin.

## Exosomes from body fluids

### Exosomes from blood

Body fluids, such as plasma, serum, urine, saliva, milk, amniotic fluid, ascites, and cerebrospinal fluid, are abundant sources of exosomes [121]. Exosomes originating from body fluids are involved in the pathophysiologic process of diabetic wound healing. For example, serum-derived exosomes promote the migration of NIH-3T3 cells and the tube formation of HUVECs to enhance angiogenesis and ECM generation to accelerate diabetic wound healing [122]. In addition, liquid biopsy of exosomes has shown promise in the diagnosis of diabetic wounds.

#### Exosomes from PRP

Platelet-rich plasma (PRP) possesses neovascularization, anti-infection, and anti-inflammatory effects [123]. However, due to the scarcity of autologous platelets, the value of PRP in clinical applications for the treatment of diabetic wounds is limited. Alternatively, PRP-derived exosomes (PRP-exos), a condensed product of PRP, have similar functional substances, but are more concentrated, less expensive, and can be easily produced without special equipment. PRP-exos enriched with various growth factors activate multiple important signals, such as PI3K/Akt, Erk, and Rho/YAP, in recipient cells to promote angiogenesis and re-epithelialization during diabetic wound healing [124]. Therefore, based on the above findings, PRP-exos are expected to be extensively used for diabetic wound healing.

MALAT1 is a frequently reported lncRNA associated with diabetic complications that plays an important role in pathogenesis and progression, and can be used as a therapeutic target and new marker for diabetes-related diseases [125]. MALAT1 expression was downregulated in FBs from patients with Diabetic foot ulcers (DFU) [126]. PRP-exos significantly upregulated MALAT1 and thus increased the viability and inhibited apoptosis and pyroptosis of FBs. Ultimately, PRP-exos promoted diabetic wound healing by promoting MALAT1/miR-374a-5p/DNMT3A/NEK7 signaling axis.

Moreover, S1P is a biologically active lipid synthesized by sphingosine kinase 1/2 and is considered to be a crucial regulator in the maintenance of endothelial cell integrity [127]. PRP-exos enriched with S1P enhanced the proliferation and migration of HUVECs and triggered angiogenesis during wound healing in diabetic mice through activation of the S1PR1/Akt/FN1 signaling pathway [128].

#### Exosomes from plasma

Plasma is widely applied in tissue engineering. Plasma-derived exosomes (plasma-exos) are released by a variety of different cells to deliver biological contents, such as cholesterol, proteins, and nucleic acids [129]. Therefore, analyzing plasma-exos and their cargo may offer new opportunities for diabetic wound healing and fluid biopsy.

Circulating miRNA-20b-5p is closely associated with diabetes, and miR-20b-5p impairs insulin signaling and alters glucose metabolism in skeletal muscle cells [130]. Xiong et al. demonstrated that exosomes isolated from the peripheral blood of type 2 diabetic patients were abundant in miR-20b-5p, which delayed diabetic wound healing by impairing the proliferation and angiogenesis of HUVECs through inhibition of the Wnt9b/β-catenin pathway [131]. Chen et al. demonstrated that exosomes isolated from diabetic patient blood (Dia-exos) inhibited proliferation and facilitated apoptosis in human skin fibroblasts (HSFs) [132]. MiR-20b-5p was highly expressed in Dia-exos, and suppressed HSF function by inhibiting VEGFA, ultimately slowing diabetic wound healing. VEGF is a critical regulator of wound healing, tissue remodeling, and collagen production [133]. In addition, miR-20b-5p inhibitors antagonized these effects, and thus miR-20b-5p inhibitors may be used as an alternative strategy to improve diabetic wound healing.

Similarly, Xiong et al. also emphasized that miR-15a-3p-enriched in Dia-exos could downregulate the NOX5/ROS signaling pathway, thus impairing angiogenesis and diabetic wound healing [134]. Xu et al. illustrated that miR-24-3p was abundant in Dia-exos [135]. In addition, miR-24-3p inhibited angiogenesis and induced apoptosis in HUVECs by targeting PIK3R3, and inhibited healing in diabetic mice. This effect could be partially reversed by the respective antagonist.

Wang et al. discovered that plasma-isolated exosomes from DFU patients (DFU-exos) had increased miR-181b-5p expression levels compared to plasma-isolated exosomes from nondiabetic patients (NDF-exos) [136]. MiR-181b-5p induced cellular senescence in HUVECs, impaired diabetic wound healing, and inhibited vasculature generation via miR-181b-5p-mediated inhibition of the Nrf2/HO-1 pathway. Furthermore, inhibitors of miRNAs could be utilized as potential targets for diabetic wound healing.

#### Exosomes from plasma after exercise

Exercise training triggers the rapid release of exosomes into the circulation, resulting in a range of beneficial effects, including enhanced endothelial function, angiogenesis and cardiovascular protection, in T2DM [137]. SOD3 is an important antioxidant enzyme that protects organisms from oxidative stress damage [138]. Abdelsaid et al. showed an upregulation of SOD3 in human plasma exosomes after a single exercise session [139]. Exosomal overexpressed SOD3 enhanced local H_2_O_2_ levels in a heparin-binding domain (HBD)-dependent manner, augmented VEGFR2 signal transduction, and promoted angiogenesis in ECs, thus accelerating diabetic wound healing. Consequently, exosome-overexpressing SOD3 could be developed as an exercise mimetic therapy to restore angiogenesis in diabetic wounds.

#### Exosomes from EPCs

EPCs are endothelial precursor cells engaged in revascularization and organizational repair of injured tissues [140]. EPCs are generated in the bone marrow and mobilized into the circulation. In diabetic wounds, EPCs are recruited to the injury site in response to signals such as various growth factors and chemokines emitted from the inflamed wound area. Once reaching the destination, EPCs differentiate into mature ECs, participating in neovascularization and, thereby supporting the wound healing process [141]. Paracrine factors secreted by EPCs can directly activate the proliferation of KCs and FBs, accelerate neovascularization, and promote diabetic wound healing. EPC-derived exosomes (EPC-exos) significantly promoted FGF-1, VEGFA, VEGFR-2, ANG-1, E-selectin, CXCL-16, eNOS, and IL-8 and inhibited MMP-9 in human microvascular endothelial cells (HMECs). The above cytokines preserve the function of HMECs, and facilitate vascularization and diabetic wound healing accompanied by suppressing inflammation. Local injection of EPC-exos strongly promoted neovascularization and wound healing in diabetic rats [142]. In addition, EPC-exos significantly enhanced the proliferation, migration, and formation of angiogenic tubules in HMECs by triggering the Erk1/2 pathway. Li et al. demonstrated that EPC-exos accelerated the healing of skin wounds in diabetic mice by facilitating the activation of HaCaTs [143]. The functions were due to the upregulation of miR-182-5p expression subsequent to the inhibition of PPARG.

HIPK2 is a serine/threonine kinase and tumor suppressor with a strong relationship to angiogenesis [144]. Xu et al. identified that miRNA-221-3p-enriched EPC-exos promoted wound healing in diabetic mice [145]. Bioinformatics analysis showed that HIPK2 is a predictive target of miR-221-3p. Subsequently, they also found that miR-221-3p could promote the viability, migration, and capillary-like tube formation of HUVECs by directly targeting HIPK2 [146].

Astragaloside IV promoted the secretion of EPC-exos containing high levels of miR-126-3p, which accelerated wound healing in rats and slowed scar formation [147]. In addition, EPC-exos with high miR-126-3p expression enhanced proliferation and migration, inhibited apoptosis and pyroptosis, and improved the vascularization ability of HUVECs by inhibiting the expression of PIK3R2 and activating the VEGF/PI3K/Akt signaling pathway.

#### Exosomes from human circulating fibrocytes

Circulating fibrocytes are a subpopulation of mononuclear phagocytes characterized by the expression of CD13, CD34, CD45, and collagen. Once formed in the bone marrow, they enter the circulation and migrate to the spleen and peripheral tissues [148]. As injury occurs, circulating fibrocytes differentiate into myofibroblasts, which contribute to wound healing and fibrosis. Geiger et al. illustrated that human circulating fibrocyte-derived exosomes (fibrocyte-exos) enhanced the migration and proliferation of KCs and promoted the secretion of collagen I, collagen III, and α-SMA in DFs, consequently leading to enhanced diabetic wound healing [149].

### Exosomes from menstrual blood

As newly discovered MSCs, menstrual blood MSCs (MenSCs) display an excellent proliferative capacity, self-renewal ability, and plasticity and are prospective stem cells in the field of tissue regeneration and aesthetic treatments [150]. Extensive literature supports the reparative potential of MenSCs in animal models of various human diseases, such as stroke, liver fibrosis, Duchenne muscular atrophy, and rotator cuff healing. Dalirfardouei et al. showed that MenSC-derived exosomes (MenSC-exos) could alleviate inflammation by inducing macrophage polarization from the M1 to M2 phenotype [151]. Besides, MenSC-exos accelerated re-epithelialization in mice through upregulation of the NF-κB p65 subunit and activation of the NF-κB signaling pathway, and improved collagen deposition and reduced scar formation by reducing the collagen I/collagen III ratios, ultimately improving diabetic wound healing.

### Exosomes from human urine

Human urine-derived stem cells (hUSCs) have biological characteristics similar to those of MSCs, including self-renewal ability, pluripotent differentiation, angiogenic paracrine effects, and immunomodulatory effects. In addition, hUSCs are easy to extract and isolate, safe, and nontumorigenic. These characteristics make hUSCs a rich cellular source for exosome production. Chen et al. found that DMBT1 is highly expressed in hUSC-derived exosomes (hUSC-exos). DMBT1 could induce high VEGFA expression in ECs through activation of the PI3K/Akt signaling pathway, thereby promoting angiogenesis in vitro, and ultimately promoting angiogenesis and wound healing in streptozotocin-induced diabetic mice [152].

### Exosomes from milk

Milk is a class of exosome-containing biofluids that can be used on an industrial scale, with advantages over any other exosome source [153]. Moreover, milk-derived exosomes (milk-exos) are cross-species tolerant without adverse immune or inflammatory reactions. Therefore, milk-exos can be used as carriers of drugs and nucleic acids and are promising candidates for diabetic wound healing. Xue et al. found that Keap1 was upregulated in diabetic mouse wounds and in HUVECs pretreated with methylglyoxal [154]. SiKeap1-loaded milk-exos (milk-exos-siKeap1) alleviated oxidative stress by increasing the nuclear localization of Nrf2 and upregulating HO-1 in HUVECs. Additionally, milk-exos-siKeap1 accelerated collagen formation and neovascularization, thereby accelerating diabetic wound healing.

## Exosomes from immune cells

### Macrophage-exos

Macrophages are an essential component of the innate immune system, for engaging in host defense, cellular regulatory functions, tissue debridement, and wound healing [155, 156]. Macrophages have two main morphologies: M1 morphology macrophages phagocytose pathogens, generate a proinflammatory state, and remove damaged cells, while M2 morphology macrophages secrete the cytokines MMP and VEGF, which accelerate tissue repair and remodeling [157]. The impaired transition of M1 to M2 macrophages is related to suppressed angiogenesis, reduced collagen deposition, and delayed diabetic wound healing. Macrophage-derived exosomes protect HUVEC function by activating Akt/VEGF signaling, and exert a potent anti-inflammatory effect by reducing the expression of TNF-α, IL-6, and MMP9, and inflammatory cell infiltration, thereby improving diabetic wound healing [158].

#### MiRNAs from macrophage-exos

Obesity contributes to the dysfunction of adipose tissue and ICs, thereby leading to inflammation, fibrosis, and restricted angiogenesis. Adipose tissue macrophage-derived exosomes in lean animals (Lean-ATM-exos) protect tissues and relieve chronic inflammation and insulin resistance [159]. Xia et al. proved that Lean-ATM-exos enriched with a large amount of miR-222-3p induced the transformation of M1 macrophages into M2 macrophages, suppressing the expression of various inflammatory mediators by targeting Bim, thereby ultimately accelerating diabetic wound healing [160].

#### Proteins from macrophage-exos

Curcumin is a low molecular weight polyphenolic compound with natural biological activities, such as anti-inflammatory, antibacterial, and antioxidant effects. Curcumin also has a good hypoglycemic effect with great potential to repair tissue damage [161, 162]. However, due to its material properties, curcumin is poorly bioavailable. Macrophage-derived exosomes have favorable biocompatibility; hence, they can serve as carriers for drug delivery [163]. Li et al. found that curcumin-carrying exosomes (Exos-cur) reduced ROS production and protected mitochondrial function in HUVECs [164]. In addition, Exos-cur inhibited inflammation and oxidative stress, and accelerated angiogenesis by activating the Nrf2/ARE signaling pathway, thereby accelerating diabetic wound healing.

## Exosomes derived from other sources

### Exosomes derived from tumor tissues

In the tumor microenvironment (TME), tumor cells can deliver biologically active molecules by generating exosomes that interact with other cells. Exosomes released by tumor cells contain a host of growth factors and are indispensable for inducing angiogenesis and cell migration both in vivo and in vitro [165]. Zhang et al. demonstrated the preliminary biosafety of oral squamous cell carcinoma tissue-derived exosomes (OSCC-Ti-exos) in mice [166]. Their team discovered that OSCC-Ti-exos increased the proliferation and migration of ECs, KCs, and FBs in vitro. In addition, OSCC-Ti-exos accelerated vascularization in vivo to promote diabetic wound healing.

### iPSC-exos

Induced pluripotent stem cells (iPSCs) are pluripotent stem cells that are usually generated from somatic cells and can be transformed into a variety of cell types [167]. With high proliferation and differentiation potential, iPSCs have strong advantages in boosting tissue regeneration [168]. Hitoshi Kobayashi et al. illustrated that iPSC-exos facilitated the regeneration of peripheral nerve fibers, angiogenesis, and migration of FBs to improve diabetic wound healing [169].

### HUVEC-exos

HUVECs are easily isolated and serve as an ideal model for studying angiogenic functions. Furthermore, HUVECs can enhance the antioxidant process, modulate the immune response, and promote cell proliferation by secreting growth factors such as IGF-1 and urocortin 1, ultimately contributing to tissue regeneration [170]. PTH is an important factor in regulating calcium-phosphorus homeostasis and bone remodeling, and can promote angiogenesis, fibrosis, and collagen deposition [171]. PTHrP-2, a derivative of PTH, has similar functions. Shen et al. identified that PTHrP-2-stimulated HUVEC-exos (PTHrP-2-HUVEC-exos) increased the proliferation and migration of HaCaTs by stimulating the PI3K/Akt signaling pathway [172]. Moreover, PTHrP-2-HUVEC-exos facilitated diabetic wound healing by accelerating epithelialization, vascularization, and collagen remodeling.

## Future perspectives and challenges

Diabetic wound healing is associated with a higher risk of infection, vascular damage, hyperactivation of inflammation, oxidative stress, and persistent failure to heal, thus constituting a current dilemma in skin disease treatment [173]. In addition, exosomes are considered an upgraded version of stem cell therapy and have enormous promise in cell-free therapies as their better efficacy and lack of immune rejection and tumorigenicity. Exosomes, represented by MSCs and skin cell sources, are emerging therapeutic candidates for improving diabetic wound healing in the four healing phases. However, some issues and challenges still need to be addressed, including exosome acquisition, drug delivery capabilities, associations with biomaterials, and prospects for clinical diagnostics.

First, no uniform standards have been established for the production, purification, isolation, storage, and characterization of exosomes. Quality control of exosomes includes quantity, yield, size, composition, and function. Subtle differences in conditions can affect the wounding efficacy of exosomes, including differences in culture conditions, pretreatment methods, cell sources, gene expression, and cell activity [174]. Exosomes are highly aggregated collections of multiple biologically active molecules, and the mechanisms by which exosomes exert their effects are likely synergistic rather than attributed to single components. Therefore, identifying and quantifying the active or function-maximizing components within exosomes can contribute to tailoring the therapeutic efficacy of exosomes or developing exosome editing strategies to achieve uniform therapeutic safety and efficacy [175]. In addition, standardized procedures for culture, extraction, isolation, and identification need to be established to ensure high-quality exosome production, content, and purity, help reduce costs, and produce comparable results between different laboratories [176].

Second, exosomes are new tools for biogenic drug delivery systems based on their high biocompatibility and stability. Compared to nanoparticles and liposomes, exosomes have low immunogenicity, low biotoxicity, and inherent abilities to immunomodulate and repair tissues [177]. However, the clinical use of natural exosomes is limited by several issues, including low functional molecule concentrations and limited repair capacity. Here, we explore three promising methods: preconditioning, genetic engineering, and integration with biomaterials, each of which offers unique advantages for optimizing exosome functionality in diabetic wound treatment.

Preconditioning refers to changing the external culture conditions or environmental factors around cells to promote the secretion of effective molecule-enriched exosomes to enhance the therapeutic effect [178]. Methods of pretreatment mainly include altering the composition of the culture medium, adjusting environmental conditions, using physicochemical inducers, or adding specific signaling molecules to induce superior capabilities. Moreover, pharmaceuticals, cytokines, and hypoxia, have been shown to significantly enhance the therapeutic efficacy of exosomes. For instance, MT pretreatment of donor cells can increase the size and yield of exosomes and significantly increase the contents of miRNAs and proteins in exosomes, thereby enhancing immunomodulation and tissue repair [179, 180].

Genetic engineering exosomes is also a promising method for overcoming the therapeutic limitations of natural exosomes. Engineered exosomes carrying various therapeutic factors are manufactured by parental cell engineering or direct exosome engineering. Parent cell engineering involves using genetic engineering techniques to transfect donor cells or to co-incubate agents with donor cells, thereby directly introducing therapeutic agents into these cells [181]. The exosomes enriched with bioactive substances are then isolated and purified from the culture supernatant. Transfection can be accomplished by chemical transfection, electroporation, or viral vectors, but practical application must consider various factors, including the type and health of the parent cells, the intrinsic properties of the cargo, and optimal experimental conditions. Acting as important regulators in tissue repair and regeneration, ncRNAs have been widely studied as cargo of carrier systems. For example, engineered miR-31-5p exosomes, derived from stably transfected HEK293 cell lines through viral transfection, serve as potent RNAi therapeutic agents by inhibiting HIF-1 and EMP-1, thereby promoting angiogenesis, fibrogenesis, and re-epithelialization to accelerate diabetic wound healing [182]. Co-incubation is commonly used with hydrophobic drugs such as curcumin. Li et al. successfully prepared Exos-cur by co-incubating curcumin with macrophages [164]. Exos-cur have strong anti-inflammatory and antioxidant effects that accelerate vascularization and diabetic wound healing. Direct exosome engineering refers to the incorporation of cargoes into exosomes through membrane permeation strategies. Current major modalities for loading cargoes into exosomes include electroporation, sonication, extrusion, pH gradient methods, coincubation, transfection, hypoallergenic dialysis, freeze-thaw cycles, and surface treatment [183]. These modalities differ in their loading efficiency and stability, and also impact the integrity of the exosome membrane. Electroporation, for instance, creates small holes in the phospholipid bilayer to allow the loading of small molecules carrying drugs or nucleotides into the exosome [184]. The membrane gap is restored automatically, and the morphology of the exosome is not altered, demonstrating the high applicability of electroporation. Yan et al. successfully synthesized miR-31-5p-loaded exosomes (milk-exos-31) to encapsulate miR-31-5p in milk-exos by electroporation [185]. MiR-31-5p in milk-exos was absorbed more efficiently and promoted proliferation, migration, and tube formation in HUVECs by downregulating HIF1AN. In addition, the milk-exos-31 promoted angiogenesis and improved diabetic wound healing in vivo. Engineering modifications are an increasingly valued procedure for potentiating exosomes, but whether exosome editing results in nonnegligible modifications or has implications for other functional components warrants further exploration.

Third, because the multiple requirements of diabetic wound management are difficult to meet with exosomes and engineered exosomes alone, the combination of exosomes and biomaterials offers new strategies and directions. Ideal biomaterials can promote the durability and stability of exosomes, enabling controlled release of exosomes in a dose- and time-dependent manner with a suitable degradation rate. For example, hydrogels show superior biocompatibility, biodegradability, and antimicrobial hemostatic ability, and can provide three-dimensional scaffolding structures such as ECM to improve exosome utilization and cell infiltration and adhesion [186]. Gingival mesenchymal stem cells (GMSCs) are stem cells isolated from the gingival lamina propria that exhibit remarkable tissue regenerative potential and immunomodulatory properties [187]. The combination of GMSC-derived exosomes with hydrogels promotes collagen deposition and remodeling and enhances vascularization and neuronal growth, thereby effectively promoting diabetic wound healing. As a natural biomaterial, the human acellular amniotic membrane (hAAM) is characterized by high biocompatibility, low immunogenicity, and resistance to infection and scarring. Xiao et al. successfully prepared a scaffold using hAAM loaded with ADSC-exos (hAAM-exos) and discovered that hAAM-exos enhanced vascularization, ECM deposition, and epithelial and dermal regeneration in vivo, eventually accelerating diabetic wound healing, which is accompanied by anti-inflammatory effects [188].

Biomaterials combined with exosomes for diabetic wound healing warrant careful consideration of their physiochemical properties and biological activity. These considerations include the biodegradability, cell compatibility, and biosafety of biomaterials, and their immunomodulatory functions on exosomes. In particular, the potential risks associated with toxic compounds and degradation products need to be evaluated to ensure the safety and effectiveness of biomaterials in treating diabetic wounds [189]. Hydrogels are commonly utilized as biomaterials in conjunction with exosomes. Compared to synthetic polymer hydrogels, natural polymer hydrogels exhibit superior biocompatibility and lower cytotoxicity, underscoring the importance of selecting suitable biomaterials for specific applications [190, 191]. Additionally, different types of collagen scaffolds, including hydrogels, sponges, and membranes, can influence the immunophenotype of MSCs, and the slowest increase in the MHC molecule expression of MSCs occurs in hydrogels [192]. Thus, different types of collagen scaffolds may also alter exosome biogenesis or interactions with immune cells, potentially affecting the ability of exosomes to regulate the immune response. Polyethylene glycol (PEG) hydrogels prepared by photopolymerization may cause damage to cells due to the introduction of toxic initiators and UV irradiation [193]. Furthermore, rapid degradation of poly-dl-lactic-co-glycolic acid (PLGA) polymer scaffolds in tissue regeneration can induce severe tissue reactions and disrupt repair processes by altering local pH levels or causing the release of cytotoxic factors [194]. Such degradation may affect the stability and functionality of encapsulated exosomes, impacting their immunomodulatory abilities and potentially leading to unintended immune responses or impaired regenerative outcomes. Therefore, further studies on the interactions between exosomes and biomaterials are needed to improve the properties of the wound-promoting effect, and to determine the optimal ratio and combination of exosomes and biomaterials.

Finally, the potential value of exosomes as diagnostic markers and targets for diabetic wounds is well understood. Early biomarkers are especially important for preventing diabetic wounds and reducing the risk of amputation and mortality. Compared with nondiabetic patients, plasma exosomal miR-20b-5p, miR-15a-3p, miR-24-3p, and miR-181b-5p were significantly upregulated in diabetic patients, and these miRNAs functionally inhibited cell viability, affected angiogenesis, and slowed diabetic wound healing, which could be reversed by corresponding miRNA inhibitors [132, 134–136]. By analyzing differences in circRNA expression profiles between DFUs and normal wounds, Tian et al. found that circRNA_072697 and circRNA_405463 could be used as biomarkers for DFUs [195]. Similarly, hsa_circ_0000907 and hsa_circ_0057362 within serum exosomes exhibited specificity in differentiating diabetic patients with DFU from those without DFU, demonstrating a high AUC in early DFU. This finding suggests their potential as serum markers for the early diagnosis of DFU [196]. However, exosomal ncRNA expression is dynamic and unstable at different stages of diabetic wound healing. In addition, a large number of highly expressed ncRNAs are expressed during the wound healing process, and individual differences in their levels are evident between the assay and baseline [197]. In addition, ncRNAs possess sophisticated regulatory mechanisms and often involve multiple targets and several signaling pathways, which may impact the specificity and sensitivity of exosomes as diagnostic markers.

## Conclusion

**Fig. 4:**
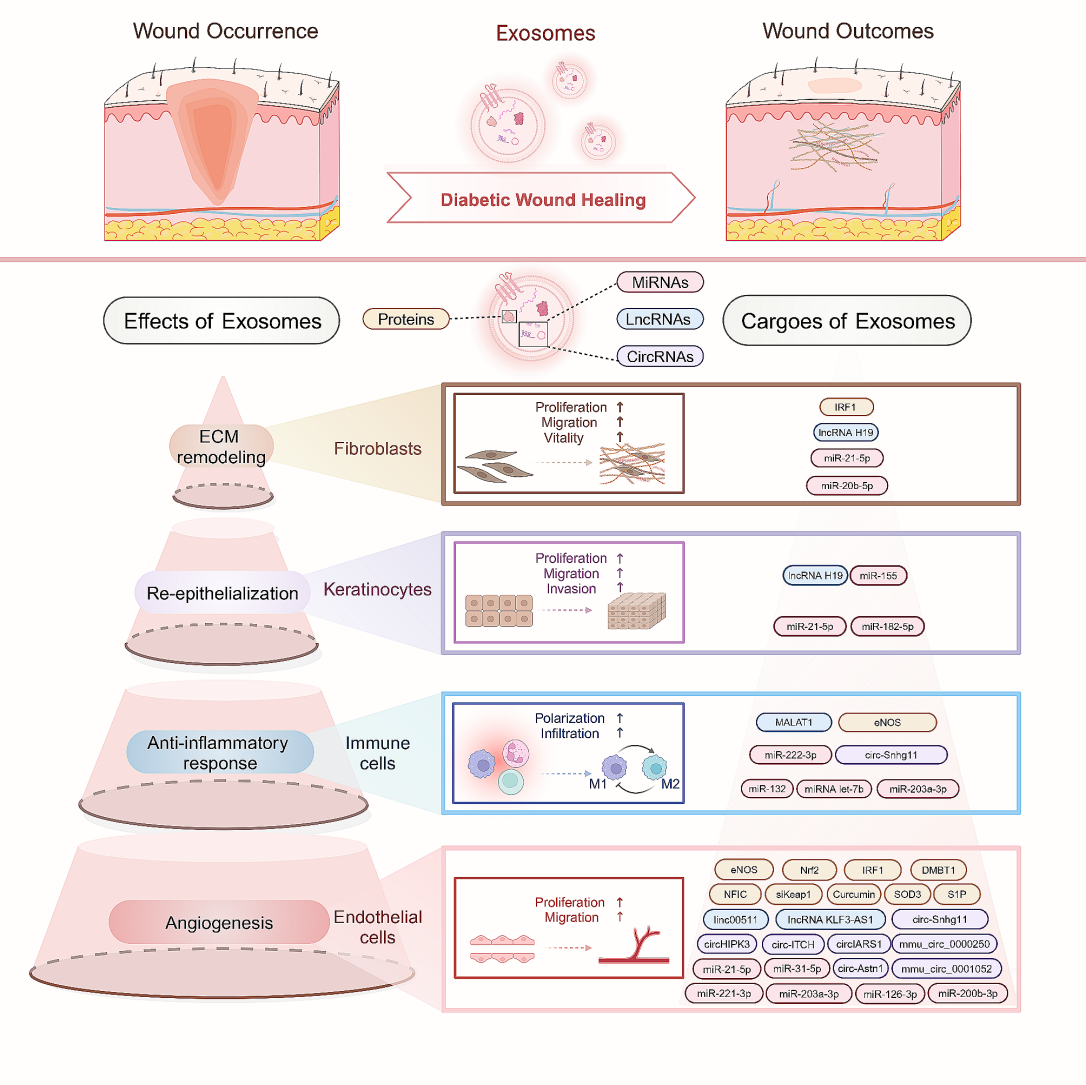
The roles and mechanisms of exosome-carried ncRNAs and proteins in diabetic wound healing. Exosome-carried ncRNAs and proteins promote the activity and function of fibroblasts, keratinocytes, immune cells, and endothelial cells in diabetic wounds. Specifically, these loaded exosomes contribute to behavior alterations of skin cells, such as proliferation, migration, polarization, invasion, and viability, consequently leading to extracellular matrix remodeling, re-epithelialization, anti-inflammatory response, and angiogenesis. These effects act synergistically and ultimately accelerate diabetic wound healing. It was created with BioRender.com

In this review, we provide a comprehensive summary and detailed analysis of all reported exosome sources and characteristics in the treatment of diabetic wounds, along with their respective mechanistic features. We aimed to develop exosome-based therapeutic strategies for diabetic wound management. In conclusion, exosomes from different sources, such as ADSC-exos, BMSC-exos, PMSC-exos, hucMSC-exos, hAMSC-exos, hAEC-exos, EPC-exos, ESC-exos, HFMSC-exos, KC-exos, DF-exos, PRP-exos, plasma-exos, fibrocyte-exos, MenSC-exos, hUSC-exos, milk-exos, macrophage-exos, OSCC-Ti-exos, iPSC-exos, HUVEC-exos, and GMSC-exos, are promising options for diabetic wound healing. These exosomes can regulate multiple cellular functions, including those of FBs, KCs, ECs, and ICs, by modulating inflammation, inhibiting oxidative stress damage, mitigating cellular senescence, promoting vascular regeneration, accelerating re-epithelialization, facilitating collagen remodeling, and reducing scarring during diabetic wound healing **(**Fig. 4; Table 1**).** These exosome candidates may serve as novel and alternative therapeutic agents for diabetic wound healing and contribute to promising strategies for the treatment of diabetic wounds. In addition, diabetic wounds are remarkably complex and involve multiple cells and bioactive factors. Therefore, the optimal dosage and route of administration must be determined to develop efficient and safe dosing strategies. Current studies on the application of exosomes are mainly based on cellular and animal experiments, which may not fully and realistically reflect the complex clinical features of diabetic wounds. Deeper basic research and clinical trials will help improve the potential therapeutic effects of exosomes with unique origins in diabetic wound healing and regeneration.

**Table 1:** The cargoes, targets, and functions of different sources of exosomes in diabetic wound healing

Exosomes type	Cargo	Target	Function	Ref.
miR-21-5p-modified hADSC-exos	miR-21-5p	Stimulating Wnt/β-catenin pathway	Stimulating proliferation and migration of KCs, and stimulating re-epithelialization, collagen remodeling, angiogenesis and vessel maturation	33
miR-132-modified ADSC-exos	miR-132	Inhibiting NF-κB pathway	Stimulating M2 polarization, attenuating local inflammation, and improving flap survival	34
linc00511-modified ADSC-exos	linc00511	Inhibiting PAQR3-induced ubiquitin degradation of Twist1	Facilitating proliferation, migration, and angiogenesis in EPCs	39
circ-Astn1-modified ADSC-exos	circ-Astn1	Inhibiting miR-138-5p/SIRT1/FOXO1 axis	Restoring angiogenic function and inhibiting apoptosis of EPCs	45
mmu_circ_0000250-modified ADSC-exos	mmu_circ_0000250	MiR-128-3p	Activating autophagy and increasing angiogenesis of EPCs	46
circ-Snhg11-modified ADSC-exos	circ-Snhg11	MiR-144-3p/HIF-1α axis	Suppressing HG-induced ECs injury and inducing M2 polarization	47
mmu_circ_0001052-modified ADSC-exos	mmu_circ_0001052	MiR-106a-5p/FGF4/p38MAPK pathway	Facilitating the vascularization of HUVECs	50
NFIC-modified hADSC-exos	NFIC	Upregulating miR-204-3p/HIPK2 axis	Promoting proliferation, migration, and angiogenesis in HUVECs	53
Nrf2-modified ADSC-exos	Nrf2	facilitating the expression of SMP30, VEGF, and VEGFR2 phosphorylation	Suppressing ROS and inflammatory cytokine expression in EPCs, and increasing granulation tissue formation, angiogenesis	57
IRF1-modified ADSC-exos	IRF1	MiR-16-5p/SP5 axis	Speeding up the proliferation and migration of FBs, and increasing vascularization of ECs	60
Atorvastatin-pretreated BMSC-exos	miR-221-3p	Inhibiting PTEN/Akt/eNOS pathway	Upregulating VEGF, PDGF, EGF, bFGF, and ANG1 in HUVECs	70
miR-155-inhibitor-loaded BMSC-exos	miR-155 inhibitor	FGF7	Accelerating angiogenesis and re-epithelialization	75
IFN-γ-pretreated BMSC-exos	miR-126-3p	SPRED1/Ras/Erk axis	Promoting angiogenic capacity	76
lncRNA KLF3-AS1-modified BMSC-exos	lncRNA KLF3-AS1	Inhibiting miR-383	Facilitating the activities of HUVECs and stimulating angiogenesis	77
lncRNA H19-modified BMSC-exos	lncRNA H19	MiR-152-3p/PTEN/PI3K/Akt axis	Enhancing proliferation and migration of FBs, and inhibiting apoptosis and inflammation	78
circ-ITCH-modified BMSC-exos	circ-ITCH	Nrf2 signaling pathway	Restoring the viability and angiogenic capacity and inhibiting ferritin deposition of HUVECs	81
LPS-pretreated hucMSC-exos	miRNA let-7b	Inhibiting TLR4/NF-κB/STAT3/Akt pathway	Promoting the transformation from THP-1 cells to M2 macrophages, and upregulating the expression of anti-inflammatory cytokines	92
Nr-CWS-pretreated hucMSC-exos	circIARS1	Activating circIARS1/miR-4782-5p/VEGFA axis	Possessing a significant proangiogenic effect	89
circHIPK3-modified hucMSC-exos	circHIPK3	Inhibiting miR-20b-5p	Accelerating vascularization	93
eNOS-modified hucMSC-exos	eNOS	Activating PI3K/Akt/mTOR or Fak/Erk1/2 pathways	Accelerating vascularization, and modulating the immune microenvironment via promoting autophagy, M2 polarization, Treg cell aggregation, and TRM cell residency	95
ESCs-exos	miR-203a-3p	SOCS3/JAK2/STAT3 pathway	Promoting M2 polarization and secretion of large amounts of VEGF, bFGF, and TGF-β	107
ESCs-exos	miR200b-3p	SYDE1/RAS/Erk pathway	Attenuating excessive autophagy-induced apoptosis of HUVECs, and promoting vasculogenesis	108
lncRNA H19-modified HFMSC-exos	lncRNA H19	Reversing the role of NLRP3 inflammasome	Promoting the proliferation and migration and inhibiting pyroptosis of HaCaTs	114
HG environment-pretreated KC-exos	LINC01435	LINC01435/YY1/HDAC8 axis	Suppressing tube formation and migratory capacity in HUVECs, and impairing angiogenesis	116
MALAT1-modified KC-exos	MALAT1	Inhibiting miR-1914-3p/MFGE8/TGFB1/SMAD3 axis	Enhancing phagocytosis and M2 polarization	117
PRP-exos	MALAT1	Promoting miR-374a-5p/DNMT3A/NEK7 axis	Increasing the viability and inhibiting apoptosis and pyroptosis of FBs	126
PRP-exos	S1P	Activating S1PR1/AKT/FN1 pathway	Enhancing the proliferation and migration of HUVECs and triggering angiogenesis	128
Dia-exos	miR-20b-5p	VEGFA	Inhibiting proliferation and facilitated apoptosis in HSFs	131
Dia-exos	miR-15a-3p	Inhibiting NOX5/ROS pathway	Impairing angiogenesis	134
Dia-exos	miR-24-3p	PIK3R3	Inhibiting angiogenesis and inducing apoptosis of HUVECs	135
DFU-exos	miR-181b-5p	Inhibiting Nrf2/HO-1 pathway	Inducing cellular senescence in HUVECs, inhibiting vasculature generation	136
plasma-exos	SOD3	Enhancing VEGFR2 signal transduction	Promoting angiogenesis in ECs	139
EPC-exos	miR-182-5p	PPARG	Promoting the proliferation and migration of and inhibiting apoptosis of HaCaTs	143
EPC-exos	miR-221-3p	HIPK2	Promoting the viability, migration, and capillary-like tube formation of HUVECs	145, 146
Astragaloside IV pretreated EPC-exos	miR-126-3p	PIK3R2/VEGF/PI3K/Akt axis	Enhancing proliferation and migration, inhibiting apoptosis and pyroptosis, and improving vascularization ability of HUVECs	147
hUSC-exos	DMBT1	Activating PI3K/Akt pathway	Promoting angiogenesis	152
miR-31-5p-modified milk-exos	miR-31-5p	HIF1AN	Promoting proliferation, migration and tube formation in HUVECs	185
siKeap1-loaded milk-exos	siKeap1	Activating Nrf2/HO-1 pathway	Alleviating oxidative stress, accelerating collagen formation and neovascularization	154
Lean adipose tissue-macrophage-exos	miR-222-3p	Bim	Inducing the transformation of M1 macrophages into M2 macrophages, and suppressing the expression of various inflammatory mediators, especially TNF-α	160
Curcumin-loaded macrophage-exos	Curcumin	Activating Nrf2/ARE pathway	Reducing ROS production and protecting mitochondrial function in HUVECs	164

**List of abbreviations:** endothelial progenitor cells, EPCs; fibroblasts, FBs; endothelial cells, ECs; keratinocytes, KCs; human skin fibroblasts, HSFs; human umbilical vein ECs, HUVECs; high glucose, HG; Reactive oxygen species, ROS.

## Data Availability

No datasets were generated or analysed during the current study.

## Abbreviations

ADSC-exos: adipose-derived stem cell-derived exosomes
BMSC-exos: bone marrow mesenchymal stem cell-derived exosomes
circRNAs: circular RNAs
DFs: Dermal fibroblasts
DM: diabetes mellitus
DFU: diabetic foot ulcers
ESCRT: endosomal sorting complex required for transport
ECs: endothelial cells
EPCs: endothelial progenitor cells
E-exos: engineered exosomes
ESCs: epidermal stem cells
EMT: epithelial-to-mesenchymal transition
Dia-exos: exosomes isolated from diabetic patient blood
ECM: extracellular matrix
EVs: extracellular vesicles
FBs: fibroblasts
GMSCs: gingival mesenchymal stem cells
HFs: hair follicles
HBD: heparin-binding domain
HG: high glucose
hAAM: human acellular amniotic membrane
hAECs: human amniotic epithelial cells
hAMSCs: human amniotic membrane mesenchymal stem cells
fibrocyte-exos: human circulating fibrocyte-derived exosomes
HDFs: human dermal fibroblasts
hMECs: human microvascular endothelial cells
HSFs: human skin fibroblasts
hUCMSCs: human umbilical cord mesenchymal stem cells
HUVECs: human umbilical vein endothelial cells
hUSCs: human urine-derived stem cells
ICs: immune cells
iPSCs: induced pluripotent stem cells
IGF1: insulin-like growth factor-1
ILVs: intraluminal vesicles
KC-exos: KC-derived exosomes
KCs: keratinocytes
lncRNAs: long-stranded non-coding RNAs
MenSCs: menstrual blood MSCs
MSCs: mesenchymal stem cells
miRNAs: micro RNAs
milk-exos: milk-derived exosomes
MVBs: multivesicular bodies
NAD: nicotinamide adenosine dinucleotide
Nr-CWS: Nocardia rubra cell wall skeleton
ncRNAs: non-coding RNAs
Nrf2: nuclear factor red factor 2-related factor
PMSCs: placenta-derived mesenchymal stem cells
plasma-exos: plasma-derived exosomes
DFU-exos: plasma-isolated exosomes from diabetic foot patients
NDF-exos: plasma-isolated exosomes from nondiabetic patients
PRP: platelet-rich plasma
PLGA: poly-dl-lactic-co-glycolic acid
PEG: polyethylene glycol
PRP-exos: PRP-derived exosomes
TEMs: tetraspanin-enriched microdomains
TME: tumor microenvironment
UBD: ubiquitin-binding domain
UTRs: untranslated regions
VEGF: vascular endothelial growth factor
α-SMA: α-smooth muscle actin
